# Stakeholders’ Expectations of Return-to-Work After Spinal Cord Injury: A 1-Year Follow-Up

**DOI:** 10.1177/15394492221097355

**Published:** 2022-05-14

**Authors:** Lisette Farias, Lisa Holmlund, Eric Asaba

**Affiliations:** 1 Karolinska Institutet, Huddinge, Sweden; 2 Karolinska Institutet, Stockholm, Sweden; 3 Stockholms Sjukhem Foundation, Sweden

**Keywords:** return to work, spinal cord injury, occupational science

## Abstract

Understanding the process of return-to-work is key to supporting people’s social participation and health after a disability. This phenomenographic study aimed to explore the expectations and ways of understanding return-to-work from the perspectives of three stakeholder types: three workers with spinal cord injuries, their employers, and an occupational therapist coordinator. Participants were interviewed twice, at 6 and 12 months, after having participated in a research-based return-to-work intervention in Sweden. A phenomenographic approach was used to analyze the data. The findings highlight how stakeholders’ different expectations prevented them from openly discussing more flexible arrangements to make return-to-work viable and sustainable. The study contributes to occupational therapy practice by raising awareness of the challenges of work reintegration. It also adds to the body of knowledge in occupational science by illuminating how normative social expectations and policy concerning work/productivity influence the return-to-work process.

## Introduction

Work has been conceptualized in diverse ways within occupational science literature, for example, as inclusive of formal and/or informal economy engagements (Dickie, 1996, 2003). Yet, return-to-work (RTW) has received less attention in occupational science and occupational therapy literature than the topic of work (Asaba et al., 2021). Occupational science scholarship regarding RTW has mainly focused on the aspects that facilitate or hinder the employment of people with disabilities, the relationship between barriers to entry to employment and concepts such as occupational injustice and occupational adaptation (e.g., Jakobsen, 2004, 2009; Soeker, 2011). In turn, the occupational therapy literature has focused on intervention studies of RTW after discharge from rehabilitation settings (e.g., Holmlund, Guidetti, Eriksson, & Asaba, 2020; Holmlund, Guidetti, Hultling, et al., 2020; Öst-Nilsson et al., 2017). Although these studies have provided valuable insights into the profession, they have focused either on practice implications or on theoretical conceptualizations of RTW. Consistent with contemporary trends in occupational science scholarship that attends to social expectations and structural factors that shape people’s possibilities to work (e.g., Aldrich & Laliberte Rudman, 2016; Asaba et al., 2021), this article highlights how stakeholders’ understandings, policy, and social expectations shape the process of RTW, which can inform the design and implementation of intervention research. As such, this article contributes to occupational therapy and builds on occupational science scholarship by exploring a phenomenon that is of interest internationally from multiple vantage points.

Due to advances in medical care over the past 20 years, the focus of rehabilitation for people with spinal cord injury (SCI) has shifted from achieving good health outcomes to long-term rehabilitation goals such as community reintegration, which includes participation in everyday life occupations, such as meaningful work (Barclay et al., 2020; Hay-Smith et al., 2013). Yet, RTW can be particularly challenging for rehabilitation teams and individuals due to the complexities inherent to the RTW process, structural barriers coupled with work accommodations, social norms and expectations, and medical complications following SCI. These challenges often lead to limited opportunities in the labor market and low employment rates after injury (Hilton et al., 2018; O’Neill & Dyson-Hudson, 2020). Limited opportunities for work reintegration can lead to long-term sick leave, which in turn can be aggravated by ambiguity in state regulations and deficiencies both in awareness for accommodation in the workplace and communication of expectations between stakeholders involved in the rehabilitation process (Coole et al., 2013; Hellman et al., 2016; Holmlund et al., 2018, Holmlund, Guidetti, Eriksson, & Asaba, 2020).

From the perspective of people with SCI, the RTW process involves uncertain everyday life situations and opportunities, partially due to fragmented support for securing and maintaining employment as part of the rehabilitation process (Bergmark et al., 2011; Holmlund et al., 2018). Scholars also emphasize the lack of immediate follow-up and support in the community after the rehabilitation process has ended (Nunnerley et al., 2013). This lack of follow-up after rehabilitation may not only impact individuals with SCI but also their coworkers, employers, and the wider community in ways that are still unclear. A limited number of qualitative studies explore RTW from the perspective of multiple stakeholder perspectives (i.e., the person with SCI and employers or rehabilitation staff). Among existing studies, most of them explore the usefulness of a specific RTW program through the experiences of people with SCI and healthcare professionals (Hay-Smith et al., 2013; Ramakrishnan et al., 2016). These experiences and perspectives are socially situated and thus can vary between country contexts and even between contexts within a country. Further studies are needed, particularly follow-up and postintervention studies, in different contexts that examine the process of work reintegration from the perspectives of those involved in the process. This knowledge can contribute to increasing understanding of RTW from multiple perspectives and how social norms and expectations shape occupations, such as meaningful work, after a disability. Therefore, the present study aims to explore the expectations and different ways in which three stakeholders (in this case workers with SCI, their employees, and an occupational therapist coordinator) understand and experience the RTW process after participating in an RTW intervention in Sweden.

## Method

Epistemologically, phenomenography assumes that the conceptions of a phenomenon may differ from one person to another depending on a given context/situation (Marton & Booth, 1997). Therefore, a phenomenographic approach was chosen as the focus of this study was to describe the variation in how stakeholders understand and experience the phenomenon of RTW postintervention from multiple perspectives (Marton & Booth, 1997). A unique contribution offered in this study is the multiple perspectives on a 1-year follow-up of RTW where employee, employer, and coordinator all have participated over time. The present study is part of a larger and interdisciplinary project investigating RTW for adults with SCI to develop and evaluate the design and feasibility of a complex intervention that can serve as a complement to current RTW practices in Sweden. The development and feasibility of the RTW intervention for adults with SCI are described in detail elsewhere (Holmlund, Guidetti, Hultling, et al., 2020). The project received ethical approval from the Regional Ethical Review Board in Stockholm, Sweden.

### Setting

SCI rehabilitation in Sweden is provided on a regional level. The first acute care that a person receives is normally at an SCI unit at a hospital, while the following inpatient care and rehabilitation are provided either in a hospital or in a rehabilitation facility. Access to SCI outpatient care, rehabilitation, and follow-up varies between regions. The healthcare services are legally required to provide medical care and rehabilitation in Sweden and should, from February 2020, also provide coordination of RTW. Implementation of coordinator services has been primarily carried out in primary health care and not yet provided in or adapted for SCI rehabilitation and outpatient care. The employer has substantial responsibility for facilitating RTW for the employee and providing healthy work environments. This includes designing a plan for RTW within 30 days of sick leave if it is assessed that the person will be sick-listed for more than 60 days.

The Swedish Social Insurance Agency (SSIA) is responsible for monitoring and coordinating the RTW process in Sweden and decides on eligibility for sick leave benefits, granting 25%, 50%, 75%, or 100% based on a certificate issued by a physician in health care. In this study, participants with SCI started 25% paid work or work trial, and after 6 to 12 months two persons increased their time to 50%. According to Swedish regulations, their part-time job needed to be equally distributed for each workday during the week (e.g., 25% work meant 2 hr per day Monday–Friday).

### Recruitment

Multiple stakeholders (i.e., persons with SCI, their employers, and an occupational therapist who coordinated the RTW intervention) were contacted via telephone by the second author. Interested individuals were provided with information about the study and provided consent to approach their employers. Informed consent was obtained prior to the interviews. Inclusion criteria for persons with SCI were: (a) had a traumatic or nontraumatic SCI, (b) between 18 and 65 years of age, (c) participated in the intervention, and (d) being able to communicate in English or Swedish. Seven persons with SCI were contacted by the second author to schedule the interviews, but only three participated in the two data collection occasions (at 6 and 12 months postintervention) for this study. The other four persons were not able to be interviewed twice due to personal and/or medical reasons.

The participants with SCI (1 woman and 2 men) did not work in the same place. They were between 33 and 62 years old and had a breadth of experiences in terms of impairment type and severity of SCI and type of employment. One person had complete paraplegia, while two had incomplete tetraplegia. Before the injury, two were employed in office work and one in manual labor. At the time of the interviews, two persons had returned to part-time work, and one was on sick leave, after having completed a work-trial. At this point, time since injury ranged between less than 1 year to 1.5 years. Two persons were living by themselves, and one was living with a partner. Two persons had children that were living at home, one full-time and the other had shared custody.

The recruited employers (2 women and 1 man) of the three participants with SCI worked in the private sector in Sweden (2 large companies, 1 small/family business) and represented varied labor sectors, such as construction, information technology, and market research. They had the principal responsibility for the RTW process and had more than 5 years of experience within their sectors. The coordinator was an occupational therapist (1 woman) that was recruited for the feasibility of the RTW intervention. She had more than 5 years of experience, employed in a specialized SCI rehabilitation facility.

### Data Generation and Analysis

The seven participants (i.e., three workers with spinal cord injuries, their employers, and a coordinator) were interviewed twice, at 6 and 12 months after having participated in a research-based RTW intervention in Sweden. A total of 14 individual semi-structured interviews were conducted in Swedish as preferred by the participants. Most of the interviews were conducted face-to-face at a location of participants’ choice, and one interview was conducted via Skype. Interviews ranged between 37 and 74 min. All interviews were audio-recorded and transcribed verbatim.

The interviews with persons having SCI were structured around everyday life, RTW training/work, and coordination between stakeholders. The interview guide consisted of questions such as: Could you tell me what has been important in your RTW process? Could you tell me about your experience with other stakeholders? The interview guide with employers consisted of questions about the RTW process (i.e., initial stage after SCI, planning, and coordination of RTW, and work training/work). Examples of questions are: Could you tell me how your employee’s new situation affected your expectations as an employer? Could you tell me what has worked well and less well during your employee’s work training/work? The interviews with the coordinator focused on her experiences of coordination and communication with other stakeholders. When appropriate, prompts were used to invite participants to provide examples to make clear the meaning of their statements.

In line with phenomenography (Marton & Booth, 1997), the starting point of the analysis was to differentiate and gather the views of the stakeholders. Using an inductive approach, the first author led the analysis process by repeatedly reading and rereading the transcripts and listening to the audio recording to gain an overall impression of the data. While reading the transcripts, the first author made notes about the potential meaning of some participant’s statements, having as a primary aim to document her reflections and preliminary interpretations concerning individual interviews and the complete dataset. The parts of the participants’ statements that were identified in accordance with the aim of the study were then highlighted, condensed, summarized, and preliminarily categorized as ways of understanding the phenomenon of RTW. After this process, the first author continued the analysis by abstracting the condensed and summarized statements and carefully comparing them to identify variations and similarities. These summarized statements were then revised among the authors and compared for accuracy. The revised statements were then grouped into preliminary descriptive categories by the first author. These preliminary categories were discussed with the other two authors to establish borders. When minor discrepancies emerged, they were discussed among authors until an agreement was reached. The three final descriptive categories were identified based on discussion among the authors. The analysis was conducted in Swedish as well as the discussions between the authors. Selected quotes used in the findings were translated by the first author after the analysis was completed and then revised by the other two authors. Two authors have Swedish as first language and good command of the English language, and one has Swedish and English as second and third languages.

## Findings

The findings are presented in three categories: (a) Expectation-experience discrepancies when reentering work; (b) working around regulations and personal needs related to the injury, and (c) feeling pressured to fulfill colleagues’ expectations and justify worth. Quotes from the three stakeholders are used to illustrate their experiences and reveal more than one way of understanding a situation in the RTW process. Pseudonyms are used in the citations.

### Expectation-Experience Discrepancies When Reentering Work

Employers often talked about people with SCI in terms of personal qualities that were evident before the injury, such as being “ambitious,” ‘hardworking,’ and “energetic.” These qualities seem to continue being important for the employees who unconsciously talked about the employee in terms of who they were before the injury. This was also evident when the employers said that they sometimes forgot that their employees may struggle with the consequences of SCI. For example, Karin (employer) noted that “sometimes you forget and expect so much and then you have to think, yes but it is like that [she has an SCI]. You forget about it sometimes because she is so positive and like an energetic person.” In this quote, it is suggested that it was challenging for some employers to adjust their work expectations to their employees’ new life situations. As Nelly (coordinator) described that the meetings with employers focused primarily on “how much the person can work” instead of what it meant to have SCI.

In contrast, people with SCI expressed a wish for employers to better understand their situation and the consequences of the injury. As expressed by Agnes (employee) “it is important [for the employer] to understand that even if you look healthy, there may be disabilities that make it difficult for you to do certain things.” This aspect was also noticed by Nelly (coordinator) who stated, “I did as best I could to really tell employers about SCI . . . to gain understanding and it may be something that you would need to repeat even more. . . I think that it was very important for the patient.” Even if the persons with SCI in this study had a favorable opinion of their employers, they expressed an unsettling expectation to perform as “before” by part of their employers. As expressed by Agnes (employee),

I notice that she [employer] has not really understood the severity of the SCI, that she only sees that you can walk or cannot walk. . . It is not so good every time then because then you get a feeling that there are expectations like “soon she is healthy, soon she will be back.”

This could indicate that employers tend to see the person with SCI as they were before the injury, and associate SCI with purely physical consequences, neglecting or remaining unaware of long-term complications. As Olof (employer) pointed out,

The starting point has always been that he should get back to what he did before the accident because his cognitive ability is not impaired so there is really no obstacle for him to be able to do it either.

Another aspect that could imbue these expectations is the notion of the “ideal employee” who focuses mostly on work, has no struggles or responsibilities outside of work, and holds strict boundaries between work and personal spheres. In the next quote, the choice of words of Olof’s (employer) could imply that he associates his employee moving from personal needs toward the needs of the workplace as his “old self” would do:

I think it is so clear that it is only now that he is really starting to become his old self because he is leaving his bubble. Before he has talked a lot about what is in the bubble, about the training . . . but now he has stepped out of the bubble, he can suddenly start talking about [company name] next-generation hardware instead.

Striving for leaving the “SCI bubble” and reentering the workplace could risk perpetuating a normative expectation of how to be at work that demands employees to keep the consequences of SCI and everyday struggles to themselves. For instance, Douglas (employee) pointed out that it is not only about the direct consequences of the injury but also about 5 to 10 other processes related to regulations, insurance, and institutions that need to be sorted out by himself to make RTW viable in practice (e.g., parking space for disabled people, housing and car modifications). Furthermore, participants with SCI described in the interviews that basic things such as taking care of their (new) body by simplifying morning routines or being well-rested before work became important foci after their injury. This finding exemplifies the different understandings between employers and persons with SCI regarding “what needs to get done” in RTW. Acknowledging differences such as these are important; Douglas (employee) says,

They do not see the other dimension, that I am completely exhausted and have a sore neck and back . . . I think they see me, like, well now he is back, so good. And, yes, I wanted to work . . . but then when I started working, damn how hard it was to work. I did not understand that.

### Working Around Regulations and Personal Needs Related to the Injury

According to the Swedish regulations, participants 25% paid work or work trial needed to be equally distributed for each workday during the week, meaning that they need to work 2 hr per day Monday to Friday. Quotes in this category include examples of how employees and employers have needed to “go around” SSIA’s regulations to make working life manageable. As Agnes (employee) described,

I do not work two hours a day as the SSIA says, but I work three days a week a little longer so that you have time to get something done. And that is good because then I have a few days to recover . . . My employer knows about it, I know about it but to the social insurance office, we write 3.75 hours per day.

Also commented by Olof (employer),

The SSIA knows that he should go to the office every day and we have ignored that because it does not work for him. Then there will be nothing at all, he does not have the strength for full days. So, the regulations are not at all adapted for his rehabilitation. So, we have talked about it and said that “yes” if the social insurance office hears from you, you work all days yes, “yes, check.”

This comment can be linked to what Nelly (coordinator) also pointed out in her interviews, “the SSIA was not as flexible perhaps as one would have liked. If you look at the person’s resources, then maybe, if you had to plan it yourself, maybe you would have done it differently.” This suggests that instead of advocating for a personalized approach to RTW, the SSIA’s regulations may hinder taking advantage of employers’ flexibility and individuals’ resources. Another example is described by Lillian (employer) who explains how she goes “around” regulations by not disclosing information to the SSIA about her financial support to her employee:

I should not pay for his gym card [he should receive free rehabilitation/training even if he is in work trial], but I know, and he knows that I pay for this because I want him to get ahead in life himself and feel independent. But it is between him and me, I do not want to inform the SSIA about this because I think they misuse their funds and do not put them where they are needed.

In this category, people with SCI, coordinator, and employers describe how they adjusted their ways of managing RTW and communication with the SSIA in ways they perceived best for the individual employee. Compared with the previous category, the complicity between stakeholders was important and expected to make the process work.

### Feeling Pressured to Fulfill Colleagues’ Expectations and Justify Worth

The persons with SCI, who had returned to paid work, described having problems completing their job obligations within their part-time work. This was partly because of different understandings about which tasks could be included within the reduced working hours, and as such, an imprecise RTW plan. Tensions arose based on previous expectations of productivity and unclear communication about the RTW plan among colleagues. For instance, most participants described a sense of pressure from their colleagues. Douglas (employee) described a situation in which he perceived that his colleagues expected help from him as they did before the injury, when he worked full-time, he said; “and then another colleague [said], can you help Emma and Per? And it was like a little pressure. And then. . . I felt a little like no [!]. Actually, he should not have asked me, so to speak.” Agnes (employee) also described a similar situation:

As soon as I come in, I feel a little stressed because I know there is an expectation that I will help them with one thing . . . And so I notice, now, in the little time I have, we have some new employees, and then like they want to pull in me and “can you look at this? can you provide your input here?” So, if I am in for three hours, then it is like spending two and a half hours helping them. And then I do not get started with my stuff, it is kind of a little tough then.

Henrik (employee) also shared a feeling about not being able to do as much as expected: “I sometimes come at eight and sometimes a little later, or shortly after eight. You have been there for a couple of hours. Then you take a cup of coffee and sit and buzz and then you have to go home (laughs).” With regard to expectations of productivity, an implicit expectation to produce “more” within their hours were linked to being very productive before the injury or because of extensive work experience and feeling responsible to justify their salaries. As Agnes (employee) described,

I cost a lot of money because I have a pretty good salary. And I feel like I have to cover my costs, plus give a surplus . . . I have to somehow show that I am worth that money. Even if I am going to work part-time because that’s how business is. Hard pressure.

Even for a participant with SCI that did not earn a high salary, a sense of pressure for “compensating” for his salary was evident. As Henrik (employee) described,

If they [employers] have the finances to fill a job that does not draw in any money, it is more like a service place then. . . Because they have to get something in, they have to be able to do something that they can draw money in. Yes, money must be drawn in.

This sense of responsibility for compensating for their salary was representative of people with SCI’s accounts in this category and is consistent with how employers seemed to make sense of people with SCI “own pressure.” As Lillian (employer) noted, “it puts pressure on the employee to perform a job . . . an employee who cannot do the job [that is expected] has a huge stress for not being able to deliver to me for whom it feels responsible.”

## Discussion

This study aimed to explore the expectations and different ways in which three stakeholders (in this case persons with SCI, their employees, and the coordinator) understand the RTW process after participating in an RTW intervention in Sweden. Understanding the variations in stakeholders’ expectations and experiences, and how these expectations and social norms influence reintegration to meaningful work, can support the development and implementation of person-centered RTW interventions. The findings provide insights into the complex interplay between social norms, workplace expectations, individuals’ needs, and policy, contributing to occupational science scholarship that argues for more situated understandings of occupations such as work (e.g., Aldrich & Laliberte Rudman, 2016; Asaba et al., 2021).

The findings illustrate how employers and persons with SCI may have expectations based on work capacity before the injury. These work-related expectations reflect social norms of work that tend to frame the RTW process as “coming back as before,” preferably full-time (Social Insurance, 2020). In this study, persons with SCI had participated in an intervention that emphasized timeliness and RTW as a gradual process in which uncertainties of everyday life and work were sorted in a dialogue between stakeholders (Holmlund, Guidetti, Hultling, et al., 2020). Yet the findings show that persons with SCI, who returned to paid part-time work, experienced expectations that were perceived as having little consideration of feelings of readiness or balance with other meaningful occupations (e.g., self-care, taking care of children, among others). For the participants with SCI, there was a sense of constant struggle against these expectations, which was at the root of wishing for more understanding among employers and colleagues. Similar expectations have been highlighted in previous research as being influential in work-related rehabilitation processes (Ahrberg et al., 2010; Nord, 2018).

According to Seing and colleagues (2015) Swedish early RTW policy is based on an idealized image of the “standard workplace,” which puts high demands for productivity on individuals and leaves little room for accommodating people in workplaces. This could explain how expectations of performing like “before” may be perpetuated. In line with this idealized image, literature indicates that working life in contemporary Western societies has been affected by increased mental demands rather than physical demands (Hellgren et al., 2008; Seing et al., 2015). This increase of mental demands may play a role in how RTW among individuals with a physical injury may be perceived as anyone else by colleagues and employers. While this can be viewed as something positive, literature shows that individuals with a physical injury tend to adopt characteristics, behaviors, and attitudes that are predominately influenced by what they believe others expect them to be (Rahim, 2010). As such, some participants in this study started to work longer hours some days to level their productivity to the standard of their workplace, and all of them described a sense of pressure to compensate for their salary by working as hard as everyone else. Yet, trying to fulfill regulations and work expectations created tensions for persons with SCI who described that their work was no longer their main occupation because they needed to handle complications from the injury and other everyday activities to make RTW “work.” Evidence suggests that managing everyday life after injury is often a priority and a condition for the viability of work (Bergmark et al., 2011; Hay-Smith et al., 2013).

Furthermore, the findings point out how individuals with SCI struggled to balance RTW in ways that follow regulations. Rigid regulations limited sustainable part-time work as understood by all stakeholders. This study shows how employers’ complicity concealed information from SSIA’s officials to enable an RTW process that functioned for the person with SCI. As such, RTW can be understood as not only framed by personal expectations and standard workplaces but also by structural barriers that risk making RTW unattainable for those who cannot work “around regulations.” Studies have pointed out rigid regulations as hinders to enacting realistic rehabilitation goals and person-centered RTW processes (Holmlund, Guidetti, Eriksson, & Asaba, 2020; Social Insurance, 2020). This study also points to how discrepancies about regulations can hinder transparent communication and integration of services (Holmlund, Guidetti, Eriksson, & Asaba, 2020). This highlights the need for a person-centered process in which consideration focuses on individuals’ needs, workplace’s conditions, type of job, among other factors that should probably lead to more realistic conditions for those in need of support (Social Insurance, 2020). Open communication between stakeholders and a person-centered process, facilitated by a coordinator could benefit dialogue around individual needs and structural conditions (Holmlund, Guidetti, Eriksson, & Asaba, 2020). Moreover, it would benefit those that may not have the support from their employers to work “around regulations,” and that need to comply with regulations that do not favor their recovery, and consequently the RTW process.

An apparent limitation of this study is the small number of participants. However, the small group of participants enabled an in-depth discussion of their experiences. The small number of participants means that the findings should be interpreted with some caution. It should also be noted that the participants in this study had participated in a larger RTW intervention with a coordinator that was an occupational therapist. Yet, the trustworthiness of this study was enhanced using triangulation of data sources (i.e., three stakeholders’ groups), keeping a trail consisting of notes, and peer debriefing among the authors. Credibility was ensured by the competencies and experience of the researchers with the group of participants, within the RTW field, and the qualitative methods employed (Shenton, 2004). To avoid losing the meaning of the quotes during translation (van Nes et al., 2010) from Swedish to English, translation of participants’ quotes was performed after the analysis was completed. The translation was conducted by the first author who has a good command of English and Swedish language and thereafter revised by the other two authors to ensure accuracy of the quotes presented. Furthermore, the study was carried out in a Western developed country, such as Sweden, and can therefore reflect particular situations that are coupled with its cultural and jurisdictional context. Nonetheless, the findings provide insights into the tensions emerging from different understandings and expectations that stakeholders had about RTW. These tensions can raise awareness of the complexity of RTW and thereby inform the design and development of interventions and follow-up studies in other Western developed countries (Young et al., 2005).

In conclusion, this study illustrates the importance of a continuous dialogue among stakeholders and regulatory bodies to solve tensions emerging from different expectations and understandings of the RTW process. This study contributes to the evidence base of occupational therapy and the advancement of occupational science by drawing attention to how the RTW process and work as a meaningful occupation are influenced by social norms and expectations. In practice, this implies that follow-up at workplaces and support over time are needed to enact collaboration and communication among stakeholders. In this work, occupational therapists could raise awareness about the influence of normative expectations about work as well as challenges in regulations, which might impede the RTW process and create unequal/absent opportunities for those involved. Moreover, further occupational science studies on how work is (mis)shaped by social norms and expectations of productivity can support the development and implementation of RTW interventions. Similarly, the relevance of and conditions for the implementation of RTW interventions can be strengthened by using community participatory research approaches in future studies to engage and build on stakeholder expertise at the community as well as regional/policy maker levels.
